# Algorithms for Investment Project Distribution on Regions

**DOI:** 10.1155/2020/3607547

**Published:** 2020-08-01

**Authors:** Mafawez Alharbi, Mahdi Jemmali

**Affiliations:** ^1^Department of Computer Science and Information, College of Science at Zulfi, Majmaah University, Al-Majmaah 11952, Saudi Arabia; ^2^Department of Computer Science, Higher Institute of Computer Science and Mathematics of Monastir, Monastir University, Monastir 5000, Tunisia

## Abstract

This paper proposes an optimization system for solving an NP-hard problem by using several new algorithms and application programs. This study aims to identify a suitable distribution of investment projects across several developed industrial regions. It is assumed that all industrial regions involved have the same economic and strategic characteristics. The problem involves a set of projects that are to be assigned across regions. Each project creates an estimated number of new jobs, and the distribution of projects can be guided by minimizing the maximum total number of newly created jobs. The problem is NP-hard one, and it is difficult to determine the most appropriate distribution. We apply scheduling algorithms in order to solve the analyzed problem. Severalheuristics are developedto obtain the appropriate distribution of newly created jobs across all regions. A branch-and-bound method is employed in order to obtain the exact solution. The performance of the algorithm is demonstrated by the experimental results for a total number of 1850 instances.

## 1. Introduction

It is important to distribute investments throughout a given country. A “good” distribution of investment resources is very important both from an economic and a social perspective since this may have a direct effect on the behavior of cities in terms of business and economic cycles. The context of this paper is the regional allocation of industrial projects. The problem is as follows: when there are several projects to be assigned across different industrial regions, how should these projects be allocated?

The solution to this problem may be based either on the principle of equity distribution or on a distribution process which is meant to minimize the maximum total number of projects to be assigned. We start with a set of projects to be undertaken in several regions, each of which represents an amount of newly created jobs and will be entirely undertaken in the chosen region. For the sake of equity, certain heuristics are applied to guide the allocation of industrial projects to different regions in order to increase the minimum total number of new jobs created [1]. To answer the research question, it is necessary to choose between minimizing the maximum total number of new jobs created and maximizing the minimum total number of new jobs created. In this study, we assume that the industrial regions involved are identical and that they share the same characteristics that affect both investment allocation and investment opportunities. For example, if the investment project consists in the creation of a marine port, then it should be allocated to a coastal region since this is an investment with specific economic characteristics. All types of investments with such specific characteristics will be excluded from this work. Heuristics and exact solutions will be used to ensure a good distribution of all investment projects to industrial regions.

The solutions to the aforementioned problem affect individuals and their everyday activities and have significant impacts on the public and private sectors and on society at large. A wide variety of resource allocation methods with different mathematical approaches to medialization have previously been used in various application areas to compute optimal solutions to the problem of the distribution of projects to several regions. The present work aims to determine the distribution of investment projects across regions by using a new objective function and newly proposed mathematical model. New solutions were developed based on the utilization of the heuristics and the optimal solutions for *P*||*C*_max_ (minimizing the maximum total completion time for parallel machines) and *P*||*C*_min_ (maximizing the minimum total completion time for parallel machines). For the first problem, which is *P*||*C*_max_, we use the heuristics and optimal solution cited by Haouari and Jemmali [2]. The respective derived bounds cited in latter work were embedded in an exact procedure which yielded an exact and effective algorithm based on a symmetry-breaking branching strategy. For the second problem, *P*||*C*_min_, a branch-and-bound (B & B) algorithm was developed by Haouari and Jemmali [1] based on enhanced lower and upper bounds. The *P*||*C*_min_ problem is also referred to as “machine covering.” A study of Walter et al. [3] also proposed an exact B & Bmethod.

The rest of this paper is structured as follows.  Section 2 discusses previous work related to the aforementioned problem, while Section 3 sets forth the analyzed problem.  Section 4 develops the lower bound for the analyzed problem, and Section 5 introduces and describes several heuristics.  Section 6 presents the proposed solution, and Section 7 describes the experimental tests applied and their results.  Section 8 concludes this paper.

## 2. Related Work

The theory of optimal control is used to tackle the unequal distribution of income between regions associated with high rates of aggregate growth for the regional allocation of investments [4]. Other applications for optimizing the allocation of investments include linear dynamic programming problems. The objective of a study by Ohtsuki [5] was to search for the optimum solution to the regional allocation problem. Bagchi et al. [6] provided a formulation for the problem of investment allocation across cities in the framework of a planning model, and a case study examining the impact of investment on the economies of Japan was carried out [7]. The study by Castells and Solé-Ollé [8] was undertaken in order to help managers make decisions, and in order to address the need for a system that provided data and information, two metaheuristic algorithms were compared, the first of which was based on a genetic algorithm and the second on the mean variance technique. The work of Salo et al. [9] focused on improved methods of resource allocation compared to the previous work that would facilitate decision analytics by helping public organizations and firms allocate resources for “lumpy” investment opportunities. Resource allocation problems are ubiquitous in governmental organizations and business. In addition to the aforementioned cited works, Luss [10] specifically addressed equitable resource allocation problems with applications in manufacturing, networks, communication, and other areas.

Ranjbar et al. [11] investigated the problem of investment project scheduling with the purpose of reducing the related costs and the amount of necessary resources and of avoiding any delay in the implementation of those projects. The project in this case consisted in a set of interrelated activities, and the authors suggested two methods for resolving the project scheduling problem, which are a genetic algorithm and relinking, whereby a schedule is created with a list of priorities. Shadrokh and Kianfar [12] suggested a genetic algorithm to solve the resource investment problem. The performance of this algorithm has been noticed to be satisfactory when compared with that of other algorithms.

Castells and Solé-Ollé [8] analyzed the main determinants of investment distribution across different regions. The expected objectives of the investment were set based on a general description of the objectives of the respective country, which focused on effective and fair trade and on the rules arising from political factors. This study employed a dynamic equation that allowed for gradual adjustment and readjustment, and the results indicated that criteria of effective trade played an important part in the geographical distribution of investment within the government infrastructure. Hartmann and Briskorn [13] provided an overview of the aforementioned extensions, which were designed based on the structure of the resource-constrained project scheduling problem (RCPSP). This paper gave a general summary related to the concepts of activity, precedence relationships, and resource constraints and also gave a summary of many lesser-known concepts. Singh [14] developed an algorithm that incorporated the priority of a project with the development of a programmed schedule, with the objective of reducing the project makespan and the penalty cost when some projects have higher priority. The proposed algorithm was shown to be an improvement of the current priority rules.

Rodrigues and Yamashita [15] developed a new precise algorithm that solved the cost of the resource availability (RACP) problem in scheduling projects. This new algorithm relied on a hybrid method in which an initial feasible solution was determined heuristically. The branching scheme resolved an RCPSP at each node, where the resources of the RACP were fixed. The new algorithm can be employed for reducing the cost of making resources available.

Sanaei et al. [16] studied the firefly algorithm, which was originally developed by Yang to optimize numerical tasks. This method was employed for solving an RCPSP. The difficulty in solving this type of problem arises from a lack of resources and priority of constraints. The algorithm begins by configuring a set of random scheduling solutions, and the primitive scheduling is then repeatedly improved using the firefly algorithm. Qi et al. [17] developed a model for multimode resources that reduced the cost of making resources available for all activities, with the aim of completing all activities of a project on schedule. When the deadline for a project is confirmed, the contractor will attempt to reduce the project cost, and this study proposes an optimization method and a useful heuristic algorithm for identifying the optimal solution. Rahmani et al. [18] proposed a new method for solving an RCPSP based on a differential search. Their approach could provide a set of preliminary schedules. Rabbani et al. [19] suggested a new approach to the multiobjective time-cost constrained RACP.

Several other problems related to scheduling problem applications can be cited in [20–23] and [24].

More recently, several works have proposed solutions for the fair distribution problem. For example, Jemmali [25] gave different approximate solutions for the project revenue assignment problem. Another study [26] focused on budget-balancing algorithms for solving the aforementioned problem and gave several algorithms. An application of load balancing of the files on different storage supports was treated in [27].

## 3. Description of the Problem

The problem analyzed in this paper can be described as follows. Given a set of *n*_*p*_ industrial investment projects *P*_1_ to be undertaken in *n*_*r*_ different industrial regions *Re*, each project *j* can be assigned to only one industrial region *i* with *i*={1,…, *n*_*r*_}. Each project *j* with *j*={1,…, *n*_*p*_} can be evaluated by applying three indicators. The number of newly created jobs is denoted by *NJ*_*j*_. The objective of this study is to ensure a suitable distribution of projects, and it is essential to define a variable that can be evaluated in order to measure the performance of the project allocation process. In the first approach, the number of new jobs created was set as the objective variable, and the overall objective was to maximize the minimum total number of new jobs created. The question then arises as to whether minimizing the maximum total number of new jobs created yields the same result.

When assigning a project *j* to an industrial region *i*, the total cumulative number of new jobs created is denoted by *t*_*j*_. The total number of new jobs created in each region *i* after the project allocation is completed is denoted by *C*_*i*_.  Example 1 illustrates the analyzed problem. The maximum number of new jobs created for all regions is denoted by *C*_max_, and the minimum number of new jobs created for all regions is denoted by *C*_min_. For each schedule, the values of *C*_*i*_ are indexed as follows: *C*_1_ ≤ *C*_1_ ≤ ⋯≤*C*_*n*_*r*__.

**Example 1 .**

We assume that, in the case of industrial investment projects, there are five new projects, i.e., *n*_*p*_ = 5. In addition, there are two regions, i.e., *n*_*r*_ = 2.  Table 1 displays the number of new jobs created for each project.

**Figure 1:**
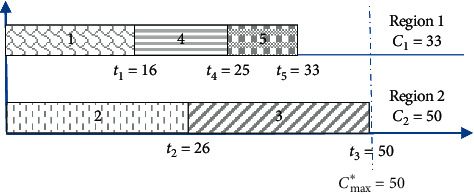
Assignment in instance 1.

Now, let us suppose that the assignment of projects to regions will be as follows. Projects 1, 4, and 5 are allocated to region 1, while projects 2 and 3 are allocated to region 2.  Figure 1 illustrates this allocation.

**Figure 2:**
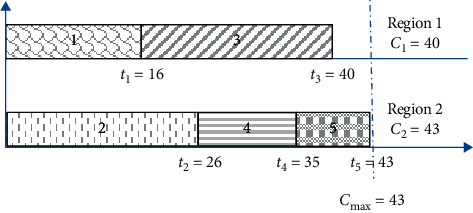
Rearrangement for instance 1.

From Figure 1, it can be noticed that there is a large gap between regions 1 and 2, which is equal to *C*_2_ − *C*_1_=17. This means that more new jobs were created in region 2 than in region 1. The objective is to decrease this gap in order to ensure a better distribution between the regions involved. There is also another arrangement that can be used in order to ameliorate the manner of project assignment with the purpose of achieving a good project distribution.  Figure 2 illustrates the new arrangement.

It can be seen from Figure 2 that the gap between regions is lower compared with the one in the first arrangement shown in Figure 1. After rearrangement, the gap is reduced to three.

**Remark 1 .**

The gap for each region *i* is given by *C*_max_ − *C*_*i*_.

**Proof**

The maximum number of new jobs created is *C*_max_. Thus, for each region, *C*_max_ − *C*_*i*_ gives the difference between the number of new jobs created in region *i* and that in the region with the maximum number of new jobs.

The indicator that can be measured to minimize the gap between regions is represented by the following formula:

(1) $$\begin{array}{c} \text{Minimize}\left[\overset{n_{r}}{\underset{i=1}{\sum }}\left(C_{\max}-C_{i}\right)\right], \end{array}$$

**Proposition 1 .**

The objective described in equation (1) can be expressed as

(2) $$\begin{array}{c} \text{Minimize}\left[n_{r}C_{\max}-\overset{n_{r}}{\underset{i=1}{\sum }}C_{i}\right], \end{array}$$

**Proof**

From equation (1), we have

(3) $$\begin{array}{c} \sum_{i=1}^{n_{r}}\left(C_{\max}-C_{i}\right)=\sum_{i=1}^{n_{r}}C_{\max}-\sum_{i=1}^{n_{r}}C_{i}=n_{r}C_{\max}-\sum_{i=1}^{n_{r}}C_{i}. \end{array}$$

By applying the minimum function to the latter result, we obtain equation (2).

Hereafter, the gap between regions that was obtained by using a given heuristic is denoted by *g*_max_^*NJ*^=*n*_*r*_*C*_max_ − ∑_*i*=1_^*n*_*r*_^*C*_*i*_. The objective of this study is to minimize *g*_max_^*NJ*^. It is worth mentioning that the constraint applied in this work consists in the fact that the project cannot be divided (i.e., the number of new jobs created by a project cannot be divided across two or more industrial regions). The optimal solution is denoted by *g*_max_^*NJ∗*^, and *LB*_max_ denotes the lower bound and *UB*_max_ the heuristic value for the *P*||*C*_max_ problem.

## 4. Lower Bounds

Two lower bounds are proposed for the analyzed problem. The first is obtained from the optimal solution to *P*||*C*_max_ and the optimal solution to *P*||*C*_min_. Hence, the balance between *P*||*C*_max_ and *P*||*C*_min_ will be considered. The second lower bound is obtained from the lower bound on *P*||*C*_max_. *C*_*max*_^*∗*^ denotes the optimal solution for *P*||*C*_max_, and *C*_*min*_^*∗*^ denotes the optimal solution for *P*||*C*_min_.

### 4.1. Min-Max-Based Lower Bound (MML)

This lower bound is determined based on the following proposition:

**Proposition 2 .**

MML=*C*_max_^*∗*^ − *C*_min_^*∗*^. The obtained value (MML) is a valid lower bound.

**Proof**

In any schedule *σ*, the value *n*_*r*_*C*_max_ − ∑_*i*=1_^*n*_*r*_^*C*_*i*_ is calculated after *C*_max_ is evaluated. This value is the difference between *C*_max_ and *C*_*i*_ for each region. This also indicates that the difference between *C*_max_ and *C*_*i*_ for all the remaining regions (excluding the region that has *C*_*i*_=*C*_max_) will be zero in this case.

In addition, *σ*(max) is the schedule obtained by applying the optimal solution to *P*||*C*_max_. In this schedule, it is obvious that no *C*_*i*_ is greater than *C*_max_, and hence, this schedule is the optimal one, with *C*_max_ being minimized. Let us suppose that the aforementioned problem may be re-envisaged as follows. Let us assume that, in the ideal case, when the two regions with *C*_max_ and *C*_min_ are excluded, the remaining regions have the same number of new jobs, which is equal to *C*_max_. In this case, the comparison is only between *C*_max_ and *C*_min_. Now, if this relaxation is applied while applying the schedule *σ*(max), the minimum value can be obtained when the value of *C*_min_ is maximized. Thus, the difference will be *C*_max_^*∗*^ − *C*_min_^*∗*^. Accordingly, *σ*(max) is then selected, which is the schedule obtained by applying the optimal solution to *P*||*C*_min_ and the relaxation; the ideal case has a difference of *C*_max_^*∗*^ − *C*_min_^*∗*^. The proposition can also be written mathematically. For any schedule, *C*_min_ ≤ *C*_min_^*∗*^ can be written as

(4) $$\begin{array}{c} -C_{\min}\geq -C_{\min}^{*}. \end{array}$$

Furthermore, in any schedule, we have

(5) $$\begin{array}{c} C_{\max}\geq C_{\max}^{*}. \end{array}$$

Combining equations (5) and (4) yields

(6) $$\begin{array}{c} C_{\max}-C_{\min}\geq C_{\max}^{*}-C_{\min}^{*}. \end{array}$$

From equation (6), it can be deduced that *C*_max_^*∗*^ − *C*_min_^*∗*^ is a valid lower bound.

### 4.2. C_max_-Based Lower Bound (CML)

This lower bound is based on the *P*||*C*_max_ one.

The C_max_-based lower bound, denoted by CML, is based on the following proposition:

**Proposition 3 .**

For a given lower bound on the *P*||*C*_max_ problem, *LB*_max_, let CML=*n*_*r*_*LB*_max_ − ∑_*j*=1_^*n*_*p*_^*NJ*_*j*_. The obtained value for CML is a valid lower bound.

**Proof**

From the objective function described in equation (2), we have ∑_*i*=1_^*n*_*r*_^(*C*_max_ − *C*_*i*_)=∑_*i*=1_^*n*_*r*_^*n*_*r*_ − ∑_*i*=1_^*n*_*r*_^*C*_*i*_=*n*_*r*_*C*_max_ − ∑_*i*=1_^*n*_*r*_^*C*_*i*_.

Furthermore, for any schedule, we have ∑_*i*=1_^*n*_*r*_^*C*_*i*_=∑_*i*=1_^*n*_*p*_^*NJ*_*j*_. Therefore,

(7) $$\begin{array}{c} n_{r}C_{max}-\sum_{i=1}^{n_{r}}C_{i}=n_{r}C_{max}=-\sum_{j=1}^{n_{p}}NJ_{j}. \end{array}$$

When considering the lower bound on the *P*||*C*_max_ problem, we have

(8) $$\begin{array}{c} LB_{\max}\leq C_{\max}. \end{array}$$

From equation (8), after multiplication by a positive integer, we obtain

(9) $$\begin{array}{c} n_{r}LB_{\max}\leq n_{r}C_{\max}. \end{array}$$

Then, ∑_*j*=1_^*n*_*p*_^*NJ*_*j*_ is subtracted from equation (9) to give

(10) $$\begin{array}{c} n_{r}LB_{\max}-\sum_{j=1}^{n_{p}}NJ_{j}\leq n_{r}C_{\max}-\sum_{j=1}^{n_{p}}NJ_{j}. \end{array}$$

From equations (7) and (10), it can be deduced that *n*_*r*_*LB*_max_ − ∑_*j*=1_^*n*_*p*_^*NJ*_*j*_ is the lower bound for the problem.

In this study, two lower bounds from the literature for the *P*||*C*_max_ problem are used for developing two versions of CML. Denoted by *LB*_*FS*_^CML^ and *LB*_*TV*_^CML^, these two lower bounds are based on ${\tilde{L}}_{FS}$ and ${\tilde{L}}_{TV}$, respectively, as described by Haouari and Jemmali [2].

## 5. Approximate Solutions

In this section, certain heuristics developed for the problem under study are presented, and these are compared to demonstrate their performance. The first heuristic is based on the nonincreasing dispatching rule, while the second is based on the nondecreasing dispatching rule. The multifit method is employed for deriving the third heuristic. In the fourth approach, we apply several times the resolution of the subset-sum problem in order to calculate the value of the heuristic. In a similar way, the fifth heuristic will solve a well-known problem iteratively; difference lies in choosing the knapsack problem which is used rather than the subset-sum problem. The last heuristic is based on rescheduling of the most charged and least charged regions to find a better result.

### 5.1. Heuristic 1: Nonincreasing Number of New Jobs Created (NIJ)

For this schedule, the total number of new jobs created for all projects is placed in a nonincreasing order. Highest *NJ*_*j*_ is then allocated to the first region on the list, i.e., the one with the lowest number of new jobs created.

**Example 2 .**

**Figure 3:**
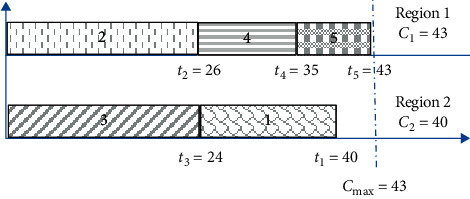
Schedule after applying NIJ in instance 1.

In this example, the same instance cited in Example 1 is considered.  Figure 3 shows the schedule after applying NIJ.

(11) $$\begin{array}{c} g_{\max}^{NJ}=2\times 43-\left(43+40\right)=86-83=3. \end{array}$$

### 5.2. Heuristic 2: Nondecreasing Number of New Jobs Created (NDJ)

In this schedule, the total number of new jobs created for all projects is placed in a nondecreasing order. Lowest *NJ*_*j*_ is then allocated to the first region on the list, i.e., the one with the lowest number of new jobs created. The schedule obtained after applying NDJ is as follows: region 1: 5,1,2 and region 2: 4,3. Thus, *g*_max_^*NJ*^=50 − 33=17.

### 5.3. Heuristic 3: Multifit Heuristic Based on the Number of New Jobs Created (MFJ)

This approximate solution is inspired by the machine job scheme presented by LEE and David Massey [28]. This method uses the bin-packing method for determining the minimum capacity that will allow all *n* new jobs created to fit into the *m* regions. For each bin capacity, the first-fit decreasing (FFD) method is employed for fitting the number of newly created jobs to the bin. Let us assume that the number of new jobs was ordered such that *NJ*_1_ ≥ ⋯≥*NJ*_*n*_*p*__. The FFD method successively assigns the project to the lowest indexed region with the necessary capacity for that respective number of new jobs. Hence, *LB*_max_=max(*NJ*_1_, *NJ*_*n*_*r*__+*NJ*_*n*_*r*_+1_, [∑_*j*=1_^*n*_*p*_^*NJ*_*j*_/*n*_*r*_]), and *UB*_max_ is the value achieved by applying the LPT heuristic for *P*||*C*_max_. Here, bin denotes the number of bins used after applying FFD, and iter is a predetermined, fixed number of iterations for FFD. In this study, iter=35.

The MFJ algorithm is illustrated in Algorithm 1.

**Example 3 .**

Assume that, in case of industrial investment projects, the number of projects is equal to 10, i.e., *n*_*p*_=10. In addition, there are two regions, i.e., *n*_*r*_=2.  Table 2 presents the number of new jobs created for each project.

For the instance given in Table 2, *UB*_max_=363 and *LB*_max_=354. *C*=[(363+354)/2]=358. Now, FFD function should be applied for a capacity equal to *C* and the 10 items represented by instance 2 after arranging them in the nonincreasing order The first bin contains items with weights {79,88,90,99}, while the second contains items with weights {21,45,55,62,83,85}. The first bin has a total load of 356, and the second has a total load of 351, giving *g*_max_^*NJ*^=356 − 351=5. Comparing this value with the results obtained using NIJ, we see that the associated *g*_max_^*NJ*^ is equal to 19. This example shows that the results obtained using MFJ are better than those obtained using NIJ. However, the difference in the number of new jobs created in regions 1 and 2 after applying MFJ is only 5.

### 5.4. Heuristic 4: Subset-Sum Problem-Based (CSS) Approach

This heuristic is based on a greedy algorithm. It is denoted by (*P*)_*k*_(*k*={1,…, *n*_*r*_ − 1}) as follows:

(12) $$\begin{array}{c} {\left(P\right)}_{k}:\left\{\begin{array}{cc} \text{minimize} & \underset{J_{j\in S_{k}}}{\sum }NJ_{j}y_{j}\\ \text{subject to} & \underset{J_{j\in S_{k}}}{\sum }NJ_{j}y_{j}\geq L\left(S_{k},n_{r}-k+1\right)y_{j}\in \left\{0,1\right\}\forall J_{j}\in S_{k}. \end{array}\right\} \end{array}$$

- *S*_1_=*P*_*I*_ and *S*_*k*=1_=*S*_*k*_/*P*_*I*_*k*__, where *P*_*I*_*k*__ is an optimal subset sum for (*P*)_*k*_(*k*=1,…, *n*_*r*_ − 1).
- *L*(*S*, *K*) denotes a valid lower bound for *P*||*C*_*max*_ on the makespan of a reduced instance, defined by *k* ≤ *n*_*r*_ regions and a subset of the number of new jobs created *S*⊆*P*_*I*_. Hence, the first region will be the number of new jobs created until *L* on (*P*)_1_ is reached. The remaining number of new jobs created in the remaining regions will constitute the second problem (*P*)_2_, which will consist in solving the new SSP until *L* is reached, and so on [29]. The subset-sum problem will be solved by using the dynamic programming algorithm developed by Pisinger [30] to solve the subset-sum problem.

### 5.5. Heuristic 5: Knapsack Problem-Based (CKS) Approach

This heuristic is based on the division of the main problem into *m* smaller problems, each of which involves scheduling of the projects to regions. In the first region, a knapsack problem is used to assign a project to region 1, and the remaining projects are assigned to the remaining regions using a further knapsack problem, and so on. For each region, a knapsack problem must be solved as follows:

(13) $$\begin{array}{c} {\left(kN\right)}_{k}:\left\{\begin{array}{cc} \text{minimize} & \underset{j\in F_{k}}{\sum }w_{j}x_{j}\\ \text{subject to} & \underset{j\in F_{k}}{\sum }NJ_{j}x_{j}\leq \left[\frac{\sum_{j\in F_{k}}NJ_{j}}{k}\right]x_{j}\in \left\{0,1\right\}\forall j\in F_{k}. \end{array}\right\} \end{array}$$

- *F*_1_=*P*_*I*_ and *F*_*k*=1_=*F*_*k*_/*P*_*I*_*k*__, where *P*_*I*_*k*__ is an optimal knapsack for (*kN*)_*k*_(*k*=1,…, *n*_*r*_ − 1).
- *w*_*j*_=(|*F*_*k*_|/*k*) × *NJ*_*j*_ − 1, where |*F*_*k*_| is the number of remaining projects, and *k* is the number of remaining regions. Hence, for the first region, the new jobs created are assigned until [∑_*j*∈*F*_1__*NJ*_*j*_/*n*_*r*_] on (*KN*)_1_ is reached. The remaining number of new jobs created and the remaining regions form the second problem (*KN*)_2_ to be solved via a new knapsack problem until [∑_*j*∈*F*_2__*NJ*_*j*_/*n*_*r*_ − 1] is reached, and so on.

### 5.6. Heuristic 6: Min-Max Iteration Heuristic (MMI)

This heuristic is based on the reformulation of *P*_2_||*C*_*max*_ as a subset-sum problem [29]. In each iteration, the most loaded region *r*_*max*_ and the least loaded region *r*_min_ were fixed, and a subset-sum problem was solved with the projects assigned to *r*_max_ and *r*_min_. This procedure was repeated several times, and the best solution was identified. The number of iterations was set to 100 based on an experimental study.

## 6. Exact Solution

The exact solution was developed using the B & B method.

**Corollary 1 .**

Minimize[*n*_*r*_*C*_max_ − ∑_*i*=1_^*n*_*r*_^*C*_*i*_] is equivalent to Minimize *C*_max_.

**Proof**

Proof. For each schedule, ∑_*i*=1_^*n*_*r*_^*C*_*i*_=∑_*j*=1_^*n*_*p*_^*NJ*_*j*_, and the objective of the studied problem can therefore be written as follows: Minimize[*n*_*r*_*C*_max_ − ∑_*j*=1_^*n*_*p*_^*NJ*_*j*_]. The total number of regions involved *m* and the summation of all new jobs created ∑_*j*=1_^*n*_*p*_^*NJ*_*j*_ are determined in advance and are fixed. This indicates that Minimize[*n*_*r*_*C*_max_ − ∑_*j*=1_^*n*_*p*_^*NJ*_*j*_] is equivalent to Minimize[*n*_*r*_*C*_max_ − ∑_*j*=1_^*n*_*p*_^*NJ*_*j*_], which is equivalent to Minimize *C*_max_.

**Lemma 1 .**

The optimal solution to the approached problem is

(14) $$\begin{array}{c} n_{r}C_{\max}^{*}-\sum_{j=1}^{n_{p}}NJ_{j}. \end{array}$$

**Proof**

Based on the Corollary 1 above, Minimize[*n*_*r*_*C*_max_ − ∑_*i*=1_^*n*_*r*_^*C*_*i*_] is equivalent to Minimize *C*_max_.

Based on the definition of *P*||*C*_*max*_, the problem is *C*_max_^*∗*^=Minimize *C*_max_. Hence, Minimize[*n*_*r*_*C*_*max*_ − ∑_*i*=1_^*n*_*r*_^*C*_*i*_] is equal to *n*_*r*_*C*_max_^*∗*^ − ∑_*j*=1_^*n*_*p*_^*NJ*_*j*_.

## 7. Experimental Results

In this section, we present the results given after the implementation of all algorithms coded in C++ using an Intel(R) Xeon(R) CPU E5-2687W v4 @ 3.00 GHz and 64 GB RAM. Several instances and statistical analyses are presented in order to illustrate the results obtained by applying the algorithms described in the previous sections. Different instances were generated which were inspired by Dell'Amico and Martello [31]. The overall instances were based on the choice of *n*, *m*, and the type of new jobs created, which is denoted by Class. The selection of the pair (*n*_*p*_, *n*_*r*_) was as follows: *n*_*p*_={10,15,50, 100,200,300} and *n*_*r*_={2,3,5,10,20,25,50}.  Table 3 shows the fixed distribution of (*n*_*p*_, *n*_*r*_).

The number of new jobs created was calculated according to the 5 distributions in the following, where *U*[] denotes the discrete uniform distribution and *N*[] the normal distribution.

Class 1: *U*[20 − 100]; Class 2: *U*[20 − 500]; Class 3: *U*[100 − 500]; Class 4: *N*[50,100]; and Class 5: *N*[20,100]. For each fixed (*n*_*p*_, *n*_*r*_, Class), 10 instances were generated, giving 1850 instances in total. The following notations were used:

- *U* indicates the best (minimum) value obtained after execution of all heuristics.
- *UB* represents the analyzed heuristic.
- Min is the number of instances for which the analyzed upper bound is equal to *U*.
- GAP_*u*_=(*UB* − *U*)/*U* represents the gap between the value obtained by applying the analyzed heuristic and the one obtained by executing the single best heuristic.
- AGAP_*u*_ is the average GAP_*u*_ for all heuristics.
- *L* is the best (maximum) value obtained after execution of all lower bounds.
- *LB* is the lower bound for the problem.
- Max is the number of instances for which the analyzed lower bound is equal to *L*.
- GAP_*l*_=(*L* − *LB*)/*LB* represents the gap between the value obtained by applying the analyzed lower bound and the one obtained by executing the single best lower bound.
- AGAP_*u*_ is the average GAP_*u*_ for all lower bounds.
- Time represents the time required to execute the heuristic or lower bound or exact solution for a corresponding instance. It is measured in seconds and is denoted by “−” if it is less than 0.001 s.
- Time_*t*_ is the average time for all lower bounds (or heuristics). It is measured in seconds and is denoted by “−” if it is less than 0.001 s.
- *NN* is the average number of nodes created in the B & B method.
- *NS* is the total number of instances that is unsolved by the B & B algorithm.

In this study, the aforementioned analyses are based on several criteria. The main criterion lies in analyzing the results obtained based on the variations in the gap and time according to the variation in *n*_*p*_, *n*_*r*_, and Class. A comparison between the lower bounds provides information on the time spent and the related gap.

The results of comparing the best lower bound and the best heuristic are significant. The average total gap between Min and Max in all 1850 instances is 0.18, with an average time of 19.99 s. This result was obtained based on the performance of the heuristic and lower bounds. Another notable result is the number of times that the lower bound was equal to the upper bound: 1684 from a total of 1850 instances (representing 91.03% of the total number of instances). This indicates that the optimal solution is reached when the lower and upper bounds are calculated. An exact solution is required for only 8.97% of the overall instances. It is worth mentioning that, for the lower bound MML which is based on the solution provided by the B & B method, if the instance is not solved by the B & B method, then the value of *C*_max_^*∗*^ is not determined. In this case, the best lower bound for *P*||*C*_*max*_ is chosen. The same procedure is followed for *P*||*C*_min_.

In Table 4, it is obvious that the gap increases when *m* increases for MML. This phenomenon is not applicable for *LB*_*TV*_^CML^ and *LB*_*FS*_^CML^. For *LB*_*TV*_^CML^, when *n*_*r*_=2, GAP_*l*_=0.06, and when *n*_*r*_=5, GAP_*l*_=1.71, but when *n*_*r*_=10, the value of the gap decreases to 0.02. The values of Time, as illustrated by Table 4, do not vary along with other values. The maximum time was 467.640 s and was reached when *n*_*r*_=50 for the MML bound. This is because this last lower bound is a consequence of solving two different problems in an exact way. The shortest time was achieved for *LB*_*TV*_^CML^ and was less than 0.001 s. The total minimum gap was 0.04 and was obtained for *n*_*r*_=2.

Other analyses are illustrated in Table 5, which shows the variation in the gap and time according to the number of new jobs created.

Time increases when *n*_*p*_ increases for *LB*_*TV*_^CML^ and *LB*_*FS*_^CML^. The smallest gap of zero is obtained for *LB*_*FS*_^CML^ when *n*_*p*_=100,  *n*_*p*_=200, and *n*_*p*_=300. The maximum gap value, which is 8.413, was obtained for MML when *n*_*p*_=300. The total maximum time was 198.545 s for *n*_*r*_=50. This explains the nonresolved exact solution provided by employing the B & B method for calculation of MML.

Table 6 presents the variation in gap and time according to Class. Table 6 shows that all classes are approximately characterized by the same magnitude of difficulty.

In Table 7, Perc indicates that the percentage of the analyzed lower bound is equal to the best value of all lower bounds for all total number of instances.

In Table 7, it can be noticed that the best lower bound, which reaches a maximum percentage of 95.89%, is *LB*_*FS*_^CML^, with an average gap of 0.22 and an average execution time of 0.114 s. The minimum percentage of approximately 38.00% is obtained by MML, with a gap of 5.87 and an execution time of 151.571 s.

Further information on the results obtained is included in Table 8.

To show how the analyzed heuristics perform, several tables of results are presented and discussed. The analyses are based on the variations in GAP_*u*_ and Time with *n*_*p*_, *n*_*r*_, and Class.  Table 9 presents the variations in gap and time based on the number of regions.

It is obvious in Table 9 that the MMI heuristic has a constant gap of zero for all values of *n*_*r*_. The heuristics NDJ and NIJ are much faster than the others, and the necessary execution time for these heuristics is lower than 0.001 s. The maximum gap of 491.61 is achieved for NDJ when *n*_*r*_=50. Furthermore, the highest time consumption of 0.018 s is seen for MMI for *n*_*r*_=50. The results show that, in almost all cases, MMI provides better results than other heuristics, but there is no dominance amongst the heuristics.  Table 10 compares GAP and Time based on *n*_*p*_.

**Figure 4:**
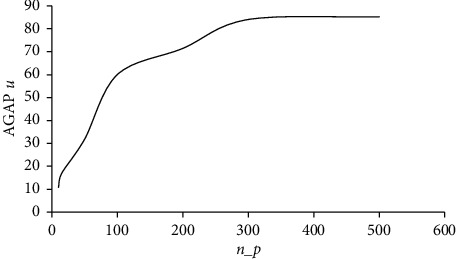
Variation of AGAP_*u*_ according to *n*_*p*_.

As it can be noticed in Table 10, the gap does not vary along with the variation of *n*_*p*_ values. The time increases according to *n*_*p*_ for CSS. The results of all executed heuristics are shown in Figure 4, where an increasing curve can be noticed.

From Table 11, it is vivid that there is a difference between the classes in terms of difficulty.

For the CSS heuristic, the greatest values for the gap can be noticed for Classes 2 and 3, meaning that these classes are more difficult to solve than the others for CSS. The same behavior was noticed for the CKS, MFJ, NDJ, and NIJ heuristics. It can be concluded that this problem is more difficult to solve for Classes 2 and 3. Furthermore, this heuristic is more time-consuming, reaching 0.585 s for Class 2.

In Table 12, it is vivid that the best heuristic is MMI, with the highest percentage of 98.2%, an average gap of zero, and an average execution time of 0.502 s. The lowest percentage that is 14.5% was obtained by NDJ, with a gap of 251.67 and a time of less than 0.001 s. Further information on the results obtained is included in Table 13.

Now, the experimental study focuses on the gap between the values of the minimum heuristic and the maximum lower bound. This requires a comparison between the best lower bound (*L*) and the best heuristic (*U*), denoted by GAP_*l*_^*u*^=(*U* − *L*)/*L*. In Table 14, the maximum gap is 0.74, which is obtained when *n*_*p*_=50, and the average time is 98.194 s. For *n*_*p*_=200, *n*_*p*_=300, and *n*_*p*_=500, the gap is zero, indicating that the optimal solution is obtained.

**Figure 5:**
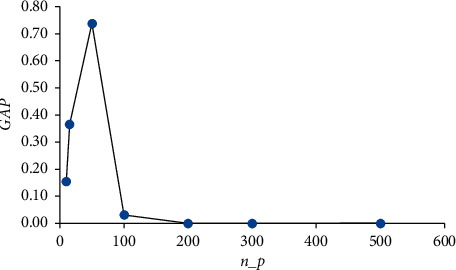
Variation of the GAP_*l*_^*u*^ value according to *n*_*p*_.

Figure 5 shows the variation in GAP_*l*_^*u*^ with the number of new jobs created, which peaks when *n*_*p*_=50 and tends asymptotically to zero when *n*_*p*_ is higher.

Table 15 illustrates the impact of the number of regions on the analyzed problem and shows that the maximum gap of 0.86 is reached when *n*_*r*_=20. However, a minimum gap of zero is reached for *n*_*r*_=2 with a reasonable time of 0.048 s and for *n*_*r*_=10 with a time of 1.262 s.

Table 16 shows the variation in GAP_*l*_^*u*^ and Time with Class. This table confirms once again that Classes 2 and 3 are more difficult to solve and require more time for that by comparison with Classes 1, 4, and 5. The maximum gap of 0.23 is obtained for Class 2, and a value of 0.28 is seen for Class 3.

The performance of the exact solution was determined by applying the B & B method, and the time limit settled at 1200 s. If the time required for solving an instance and obtaining the exact solution exceeded this time limit, the resolution process was stopped, and it was noted that the respective instance remained unresolved.

Table 17 presents the results obtained by employing the B & B method based on the number of new jobs created. It is vivid that the number of unsolved instances is higher when *n*_*p*_=50 with *NS*=48 and for *n*_*p*_=100 with *NS*=20. The corresponding numbers of nodes are 3,019,072 and 725,107, respectively. However, when the number of new jobs created is greater than 100, the problem is solved rapidly and easily, with a time of 6.572 s for *n*_*p*_=500 as compared with a time of 195.614 s for *n*_*p*_=50. A total of 70 instances remained unresolved when employing the B & B method which represents 3.78% of the total number of instances that is 1850.

Table 18 shows that the instances that remained unresolved when using the exact method are concentrated for *n*_*r*_=20, *n*_*r*_=25, and *n*_*r*_=50. The maximum value of Time was observed when *n*_*r*_=20 and was equal to 179.407 s.

Table 19 shows the differences between classes for the exact solution *g*_max_^*NJ∗*^.

In Table 19, it obvious that the maximum number of unsolved instances was obtained for Class 3 with *NS*=20. Further results obtained by employing the B & B method is presented in Table 20.

The results included in Tables 17 and 18 motivated an investigation of *n*_*p*_ and *n*_*r*_ in order to determine the instances which are the most difficult to solve. A study based on the ratio *n*_*p*_/*n*_*r*_ was carried out. For any ratio *n*_*p*_/*n*_*r*_ for the given instances amongst the total number of 1850 instances, the unsolved instances *NS*, the average number of nodes *NN*, and the average value of Time were calculated.

For the total number of instances given at the beginning of this section, there were 22 different values of the ratio *n*_*p*_/*n*_*r*_. The results are illustrated in Table 21. Indeed, Table 21 shows the performance indicators used for measuring the results obtained by the B & B method for variations in the ratio *n*_*p*_/*n*_*r*_.

**Figure 6:**
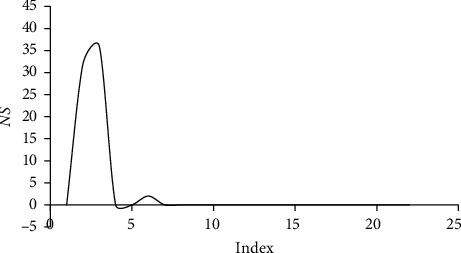
Variation of *NS* according to *n*/*m*.

The maximum average time of 870.017 s was obtained for (*n*_*p*_/*n*_*r*_)=2.5. It is obvious that the unsolved instances were concentrated for (*n*_*p*_/*n*_*r*_)=2 and (*n*_*p*_/*n*_*r*_)=2.5. It can therefore be concluded that these types of instances are the most difficult to solve.  Figure 6 shows the concentrated unsolved instances for index 2 and index 3, which are represented in Table 21 and referred to as (*n*_*p*_/*n*_*r*_)=2 and (*n*_*p*_/*n*_*r*_)=2.5, respectively.

The ratio *n*_*p*_/*n*_*r*_ represents the economic scale that can define the hardness of the problem to give a fair distribution of the projects to create new jobs on the regions. The experimental results give an appreciated gap between regions using heuristics. On the contrary, the exact method gives the optimal solution of the problem analyzed. Reducing the gap related to the new job created between regions decreases the unemployment rate in the internal cities of the country and strengthens regional development.

## 8. Conclusion

The problem analyzed above is of important practical interest as it models real-life situations in the field of economics and finance, such as equal distribution between different regions, and attempts to minimize the differences between them in terms of levels of investment. The solution presented in this paper will therefore be useful from a practical point of view. From a theoretical perspective, the approached problem is a challenging one as it is difficult to solve; thus, its resolution will enhance the theoretical knowledge in this field. The analyses and resolution of this problem may therefore have an important impact on the economy and the industrial sector. In this paper, a comprehensive literature review on resource allocation and optimization studies was presented, and the theory of optimization was applied to a real-life case study to illustrate the importance of this practice. The theoretical and practical analyses are closely related. This study was applied to the industrial sector, and the results achieved by applying heuristics and an exact solution to the analyzed problem demonstrate the importance of optimization applied on the project distribution field. The results contribute to reducing the difference between various industrial regions and to achieving equity among them. More other branching methods can be developed to ameliorate the execution time of the exact solution. The proposed solutions in this paper can be applied to several fields.

## Figures and Tables

**Algorithm 1:**
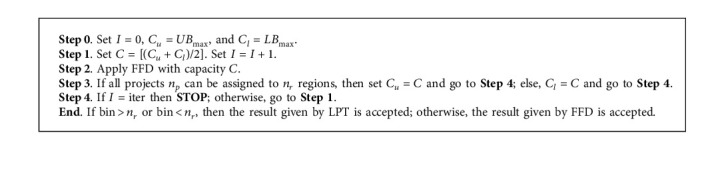
MFJ algorithm.

**Table 1:** New jobs created in instance 1.

*j*	1	2	3	4	5
*NJ*_*j*_	16	26	24	9	8

**Table 2:** New jobs created in instance 2.

*j*	1	2	3	4	5	6	7	8	9	10

*Nj_j_*	62	88	55	21	90	45	99	79	83	85

**Table 3:** Choice of (*n*_*p*_, *n*_*r*_).

*n*_*p*_	*n*_*r*_
10	2,3,5
15	2,3,5,10
50	2,3,5,10,20,25
100,200,300	2,3,5,10,20,25,50

**Table 4:** Behavior of GAP_*l*_ and Time according to *n*_*r*_.

*n*_*r*_	MML		*LB*_*TV*_^CML^		*LB*_*FS*_^CML^		Total	
	GAP_*l*_	Time	GAP_*l*_	Time	GAP_*l*_	Time	AGAP_*l*_	Time_*t*_
2	0.00	0.268	0.06	0.001	0.06	0.002	0.04	0.090
3	0.28	1.104	1.24	—	1.23	0.001	0.92	0.369
5	1.05	1.464	1.71	0.038	0.48	0.046	1.08	0.516
10	3.77	7.340	0.02	0.044	0.00	0.060	1.27	2.481
20	7.72	419.415	0.12	0.084	0.10	0.140	2.65	139.880
25	11.16	316.203	0.77	0.097	0.00	0.183	3.98	105.495
50	23.03	467.640	2.55	0.201	0.00	0.471	8.53	156.104

**Table 5:** Behavior of GAP_*l*_ and Time according to *n*_*p*_.

*n*_*p*_	MML		*LB*_*TV*_^CML^		*LB*_*FS*_^CML^		Total	
	GAP_*l*_	Time	GAP_*l*_	Time	GAP_*l*_	Time	AGAP_*l*_	Time_*t*_
10	0.53	0.814	3.94	—	1.37	0.001	1.95	0.272
15	1.12	3.326	1.06	—	0.82	0.001	1.00	1.109
50	3.12	595.614	0.74	0.001	0.09	0.019	1.31	198.545
100	7.70	294.113	1.70	0.006	0.00	0.063	3.13	98.060
200	7.68	27.450	0.00	0.031	0.00	0.092	2.56	9.191
300	8.43	6.123	0.00	0.088	0.00	0.165	2.81	2.125
500	8.29	8.763	0.03	0.249	0.01	0.360	2.78	3.124

**Table 6:** Variation of GAP_*l*_ and Time according to Class.

Class	MML		*LB*_*TV*_^CML^		*LB*_*FS*_^CML^		Total	
	GAP_*l*_	Time	GAP_*l*_	Time	GAP_*l*_	Time	AGAP_*l*_	Time_*t*_
1	5.97	149.872	0.24	0.052	0.05	0.096	2.09	50.007
2	5.72	157.569	1.10	0.052	0.33	0.109	2.38	52.577
3	5.86	177.234	1.85	0.065	0.52	0.118	2.74	59.139
4	5.99	144.349	0.29	0.049	0.13	0.103	2.14	48.167
5	5.83	128.831	0.69	0.086	0.04	0.142	2.19	43.019

**Table 7:** Comparison between lower bounds.

	MML	*LB*_*TV*_^CML^	*LB*_*FS*_^CML^
Max	703	1676	1774
Perc	38.00%	90.59%	95.89%
GAP_*l*_	5.87	0.84	0.22
Time	151.571	0.061	0.114

**Table 8:** Lower bound details.

*n*_*p*_	*n*_*r*_	MML		*LB*_*TV*_^CML^		*LB*_*FS*_^CML^	
		GAP_*l*_	Time	GAP_*l*_	Time	GAP_*l*_	Time
10	2	0.00	0.383	0.44	—	0.44	—
	3	0.14	0.961	3.67	—	3.66	0.001
	5	1.45	1.097	7.72	—	0.00	0.002

15	2	0.00	0.397	0.00	—	0.00	—
	3	0.38	1.188	0.04	—	0.04	0.001
	5	0.05	2.695	4.17	—	3.26	0.002
	10	4.06	9.025	0.03	—	0.00	0.003

50	2	0.00	0.272	0.00	—	0.00	0.001
	3	0.32	1.165	0.00	0.001	0.00	0.001
	5	1.22	1.778	0.00	0.001	0.00	0.003
	10	3.82	3.935	0.00	0.001	0.00	0.008
	20	2.95	2070.017	0.61	0.002	0.51	0.027
	25	10.39	1496.519	3.85	0.001	0.00	0.076

100	2	0.00	0.239	0.00	—	0.00	0.001
	5	0.90	1.719	0.00	0.002	0.00	0.007
	10	3.58	3.447	0.00	0.004	0.00	0.016
	20	8.52	7.816	0.00	0.007	0.00	0.038
	25	12.79	60.180	0.00	0.008	0.00	0.054
	50	20.39	1691.275	10.22	0.013	0.00	0.260

200	2	0.00	0.243	0.00	—	0.00	0.002
	5	1.30	1.177	0.00	0.014	0.00	0.018
	10	4.02	3.065	0.00	0.023	0.00	0.043
	20	8.04	6.714	0.00	0.036	0.00	0.089
	25	10.46	8.638	0.00	0.041	0.00	0.115
	50	22.27	144.866	0.00	0.073	0.00	0.287

300	2	0.00	0.176	0.00	—	0.00	0.002
	5	1.40	1.134	0.00	0.039	0.00	0.045
	10	3.06	2.836	0.00	0.065	0.00	0.088
	20	10.98	6.601	0.00	0.104	0.00	0.178
	25	11.00	8.046	0.00	0.122	0.00	0.219
	50	24.14	17.942	0.00	0.198	0.00	0.458

500	2	0.00	0.163	0.00	0.006	0.00	0.009
	5	1.03	0.648	0.08	0.206	0.08	0.245
	10	4.10	21.731	0.10	0.172	0.00	0.204
	20	8.10	5.927	0.00	0.273	0.00	0.368
	25	11.14	7.631	0.00	0.315	0.00	0.453
	50	25.34	16.48	0.00	0.523	0.00	0.881

**Table 9:** Behavior of GAP_*u*_ and Time according to *n*_*r*_.

*n*_*r*_	NDJ		NIJ		MFJ		CSS		CKS		MMI	
	GAP_*u*_	Time	GAP_*u*_	Time	GAP_*u*_	Time	GAP_*u*_	Time	GAP_*u*_	Time	GAP_*u*_	Time
2	68.61	—	5.45	—	2.72	0.002	0.00	—	0.00	—	0.00	0.012
3	116.23	—	19.83	—	12.15	—	0.54	—	0.43	—	0.00	0.008
5	166.34	—	5.40	—	4.06	0.003	0.56	—	0.76	0.001	0.00	0.013
10	302.96	—	18.41	—	12.36	0.004	0.40	0.001	0.13	0.001	0.00	0.014
20	335.60	—	21.92	—	14.52	0.005	2.73	0.001	3.17	0.003	0.00	0.014
25	371.27	—	24.46	—	15.66	0.005	8.18	0.001	1.32	0.003	0.00	0.014
50	491.61	—	37.99	—	24.19	0.006	8.11	0.003	2.34	0.007	0.00	0.018

**Table 10:** Behavior of GAP_*u*_ and Time according to *n*_*p*_.

*n*_*p*_	NDJ		NIJ		MFJ		CSS		CKS		MMI	
	GAP_*u*_	Time	GAP_*u*_	Time	GAP_*u*_	Time	GAP_*u*_	Time	GAP_*u*_	Time	GAP_*u*_	Time
10	40.59	—	8.39	—	3.79	0.000	0.55	—	0.85	—	0.000	0.005
15	64.68	—	10.92	—	6.98	0.001	1.01	—	1.18	—	0.000	0.008
50	128.78	—	14.88	—	9.43	0.001	2.48	0.001	3.69	0.001	0.000	0.010
100	252.70	—	23.27	—	15.32	0.002	7.51	0.001	1.41	0.002	0.000	0.007
200	320.66	—	19.21	—	12.87	0.004	4.41	0.001	0.19	0.003	0.000	0.016
300	383.54	—	22.02	—	14.39	0.006	0.35	0.001	0.00	0.003	0.000	0.024
500	402.88	—	14.10	—	9.06	0.010	0.12	0.002	0.02	0.004	0.000	0.017

**Table 11:** Behavior of GAP_*u*_ and Time according to *n*_*p*_.

*n*_*p*_	NDJ		NIJ		MFJ		CSS		CKS		MMI	
	GAP_*u*_	Time	GAP_*u*_	Time	GAP_*u*_	Time	GAP_*u*_	Time	GAP_*u*_	Time	GAP_*u*_	Time
1	101.69	—	6.09	—	4.40	0.005	0.82	0.001	0.89	0.002	0.001	0.378
2	432.12	—	24.80	—	12.79	0.002	4.68	0.001	1.36	0.002	0.001	0.585
3	360.65	—	21.86	—	17.16	0.006	4.96	0.001	1.24	0.002	0.001	0.566
4	263.52	—	19.53	—	9.31	0.001	0.91	0.001	0.89	0.002	0.001	0.510
5	100.38	—	12.82	—	11.16	0.006	1.46	0.001	0.91	0.002	0.001	0.473

**Table 12:** Comparison between the heuristics overall in 1850 instances.

	NDJ	NIJ	MFJ	CSS	CKS	MMI
Min	268	605	680	1507	1558	1817
perct	14.5%	32.7%	36.8%	81.5%	84.2%	98.2%
GAP_*u*_	251.67	17.02	10.97	2.57	1.06	0.00
Time	—	—	0.004	0.001	0.002	0.502

**Table 13:** Detailed heuristics for all 1850 instances.

*n*_*p*_	*n*_*r*_	NDJ		NIJ		MFJ		CSS		CKS		MMI	
		GAP_*u*_	Time	GAP_*u*_	Time	GAP_*u*_	Time	GAP_*u*_	Time	GAP_*u*_	Time	GAP_*u*_	Time
10	2	76.10	—	13.86	—	5.04	0.001	0.00	—	0.00	—	0.00	0.004
	3	41.90	—	11.19	—	6.32	—	0.88	—	0.94	—	0.00	0.005
	5	3.77	—	0.11	—	0.00	—	0.76	—	1.62	—	0.00	0.006

15	2	69.34	—	15.40	—	8.78	—	0.00	0.001	0.00	—	0.00	0.007
	3	162.32	—	23.55	—	16.23	0.001	0.74	—	0.36	—	0.00	0.008
	5	24.83	—	4.73	—	2.93	0.001	3.17	—	3.67	—	0.00	0.008
	10	2.22	—	0.00	—	0.00	0.001	0.14	—	0.67	0.001	0.00	0.009

50	2	54.24	—	2.40	—	1.04	0.001	0.00	0.001	0.00	—	0.00	0.009
	3	144.48	—	24.75	—	13.92	—	0.00	—	0.00	—	0.00	0.010
	5	176.76	—	12.13	—	10.35	0.002	0.00	—	0.00	0.001	0.00	0.011
	10	355.62	—	41.15	—	27.37	0.001	1.50	0.001	0.00	0.001	0.00	0.011
	20	35.12	—	8.75	—	3.91	—	10.51	0.001	15.86	0.002	0.00	0.010
	25	6.48	—	0.11	—	0.00	0.002	2.84	0.001	6.30	0.001	0.00	0.008

100	2	59.04	—	2.16	—	1.36	0.003	0.00	—	0.00	—	0.00	0.006
	5	259.63	—	9.71	—	7.49	0.003	0.00	0.001	0.00	0.001	0.00	0.005
	10	436.92	—	37.01	—	24.13	0.002	0.00	0.000	0.00	0.001	0.00	0.004
	20	413.24	—	48.26	—	32.55	0.002	3.12	0.001	0.00	0.002	0.00	0.006
	25	339.63	—	42.27	—	26.38	0.002	38.06	0.001	0.28	0.003	0.00	0.009
	50	7.77	—	0.24	—	0.00	0.003	3.87	0.003	8.20	0.005	0.00	0.012

200	2	84.48	—	2.36	—	1.64	0.003	0.00	0.001	0.00	—	0.00	0.013
	5	218.62	—	4.66	—	3.24	0.004	0.00	—	0.00	0.001	0.00	0.015
	10	240.44	—	8.97	—	6.20	0.004	0.00	0.001	0.00	0.001	0.00	0.016
	20	381.60	—	25.17	—	17.86	0.004	0.00	0.001	0.00	0.003	0.00	0.017
	25	541.64	—	35.05	—	22.27	0.004	0.00	0.001	0.00	0.003	0.00	0.018
	50	457.16	—	39.03	—	25.99	0.003	26.45	0.003	1.17	0.007	0.00	0.019

300	2	80.72	—	1.00	—	0.40	0.004	0.00	—	0.00	—	0.00	0.021
	5	204.78	—	3.94	—	3.02	0.006	0.00	0.001	0.00	0.001	0.00	0.023
	10	431.95	—	12.70	—	8.71	0.006	0.00	0.001	0.00	0.002	0.00	0.023
	20	269.86	—	11.30	—	8.03	0.006	0.00	0.001	0.00	0.003	0.00	0.023
	25	458.49	—	26.13	—	16.55	0.006	0.00	0.001	0.00	0.004	0.00	0.024
	50	855.45	—	77.06	—	49.65	0.007	2.13	0.003	0.00	0.007	0.00	0.028

500	2	56.32	—	0.96	—	0.76	0.005	0.00	0.001	0.00	—	0.00	0.023
	5	275.98	—	2.53	—	1.38	0.008	0.00	0.001	0.00	0.001	0.00	0.021
	10	350.62	—	10.65	—	7.79	0.010	0.74	0.001	0.10	0.002	0.00	0.018
	20	578.18	—	16.10	—	10.24	0.011	0.00	0.002	0.00	0.004	0.00	0.016
	25	510.10	—	18.75	—	13.09	0.011	0.00	0.002	0.00	0.005	0.00	0.010
	50	646.07	—	35.62	—	21.11	0.013	0.00	0.004	0.00	0.010	0.00	0.014

**Table 14:** Variation of GAP_*l*_^*u*^ according to *n*_*p*_.

*n*_*p*_	GAP_*l*_^*u*^	Time
10	0.15	0.274
15	0.37	0.547
50	0.74	98.194
100	0.03	48.519
200	0.00	4.609
300	0.00	1.119
500	0.00	1.632

**Table 15:** Variation of GAP_*l*_^*u*^ according to *n*_*r*_.

*n*_*r*_	GAP_*l*_^*u*^	Time
2	0.00	0.048
3	0.15	0.186
5	0.21	0.268
10	0.00	1.262
20	0.86	87.197
25	0.03	53.175
50	0.05	78.235

**Table 16:** Variation of GAP_*l*_^*u*^ according to Class.

Class	GAP_*l*_^*u*^	Time
1	0.14	25.035
2	0.23	26.337
3	0.28	29.617
4	0.18	24.126
5	0.06	21.550

**Table 17:** Behavior of *NS*, *NN*, and Time according to *n*_*p*_.

*n*_*p*_	*g*_max_^*NJ∗*^		
	*NS*	*NN*	Time
10	0	54	0.805
15	0	1712	1.239
50	48	3,019,072	195.614
100	20	725,107	86.107
200	2	46,388	15.409
300	0	1	6.108
500	0	7778	6.572

**Table 18:** Behavior of *NS*, *NN*, and Time according to *n*_*r*_.

*n*_*r*_	*g*_max_^*NJ∗*^		
	*NS*	*NN*	Time
2	0	9	0.267
3	0	427	1.098
5	0	811	1.390
10	0	7778	3.853
20	36	2,344,630	179.407
25	12	1,278,257	66.588
50	22	1,157,241	149.563

**Table 19:** Behavior of *NS*, *NN*, and Time according to Class.

Class	*g*_max_^*NJ∗*^		
	*NS*	*NN*	Time
1	15	763,866	52.394
2	14	510,573	51.717
3	20	1,008,239	69.979
4	13	582,937	46.725
5	8	215,072	31.380

**Table 20:** Branch-and-bound result.

*n*_*p*_	*n*_*r*_	*g*_max_^*NJ*^*∗*^^		
		*NS*	*NN*	Time
10	2	0	26	0.382
	3	0	131	0.956
	5	0	6	1.078

15	2	0	33	0.397
	3	0	1149	1.175
	5	0	5665	1.957
	10	0	1	1.428

50	2	0	1	0.272
	3	0	1	1.164
	5	0	1	1.778
	10	0	1	3.934
	20	36	11,723,146	870.017
	25	12	6,391,280	296.519

100	2	0	1	0.238
	5	0	1	1.718
	10	0	1	3.447
	20	0	1	7.813
	25	0	1	12.151
	50	20	4,350,639	491.275

200	2	0	1	0.243
	5	0	1	1.176
	10	0	1	3.064
	20	0	1	6.705
	25	0	1	8.623
	50	2	278,324	72.643

300	2	0	1	0.176
	5	0	1	1.132
	10	0	1	2.829
	20	0	1	6.586
	25	0	1	8.030
	50	0	1	17.896

500	2	0	1	0.163
	5	0	1	0.888
	10	0	46,664	8.418
	20	0	1	5.914
	25	0	1	7.615
	50	0	1	16.44

**Table 21:** Ratio *n*_*p*_/*n*_*r*_ results of branch-and-bound analysis.

Index	*n*_*p*_/*n*_*r*_	*NS*	*NN*	Time
1	1.5	0	1	1.428
2	2	32	3,580,642	262.957
3	2.5	36	11,723,146	870.017
4	3	0	5665	1.957
5	3.33	0	131	0.956
6	4	2	139,162	42.397
7	5	0	294	3.326
8	6	0	1	17.896
9	7.5	0	33	0.397
10	8	0	1	8.623
11	10	0	1	7.092
12	12	0	1	8.030
13	15	0	1	6.586
14	16.66	0	1	1.164
15	20	0	1	4.132
16	25	0	1	3.093
17	30	0	1	2.829
18	40	0	1	1.176
19	50	0	23,332	4.328
20	100	0	1	1.132
21	150	0	1	0.176
22	250	0	1	0.163

## Data Availability

All the data used to support the findings of this study are included within the article.
